# Comment on “HAX1 Augments Cell Proliferation, Migration, Adhesion, and Invasion Induced by Urokinase-Type Plasminogen Activator Receptor”

**DOI:** 10.1155/2013/782327

**Published:** 2013-05-22

**Authors:** Alicja Trebinska, Ryszard Konopinski, Ewa A. Grzybowska

**Affiliations:** Cancer Center Institute, Roentgena 5, 02-781 Warsaw, Poland

Multifunctional HAX-1 protein is involved in the regulation of several key processes like calcium homeostasis, apoptosis, and cell migration. Clarification of its role in these or other processes and its molecular mechanisms of function remains to be established. Mekkawy et al. [1] report HAX-1 influence on cell proliferation, migration, adhesion, and invasion induced by the urokinase-type plasminogen activator receptor (uPAR), as a followup of the previous study, in which the authors demonstrated uPAR-HAX-1 interaction [2]. We would like to report our serious concerns about the HAX-1 expression system used in the study of Mekkawy et al., 2012 [1]. The authors use either pGEM-3Zf(+)HAX1 or pGEM-3Zf(+) as an empty vector to transfect human cell lines. These vectors are intended for *in vitro* transcription and propagation in bacteria; they are not mammalian expression vectors and do not possess the appropriate promoter sequences. pGEM-HAX-1 used in this study was obtained from Maria Olsson and is described in the work of Dufva et al. [3] as a vector generated for *in vitro* transcription by cloning HAX-1 coding region into pGEM-3Zf(+). Unless the vector was modified, we do not see the possibility of HAX-1 expression, other than endogenous, in the studied cell lines. Furthermore, no evidence of HAX-1 overexpression was provided (e.g., Western blot). Regarding these concerns, we consider the results indicating HAX-1 influence meaningless. 
